# Cyberbullying and Problematic Internet Use as Correlates of Eating-Disorder Symptomatology and Health-Related Quality of Life in Women Under Specialized Care

**DOI:** 10.3390/healthcare14040476

**Published:** 2026-02-13

**Authors:** Isabel Panea-Pizarro, Sonia Prieto-de Benito, Andrés Ignacio García-Notario, María Aranzazu Sánchez-Calabuig, Carmen López-Sánchez, Virginio García-López, Fidel López-Espuela

**Affiliations:** 1 Mental Health Department, Hospital General Universitario, 13001 Ciudad Real, Spain; isabelpanea.pizarro@hotmail.com; 2 Hospital Clínico Universitario de Valladolid, Av. Ramón y Cajal, 3, 47003 Valladolid, Spain; sprietodb104@gmail.com; 3 Facultad de Ciencias Biomédicas y de la Salud, Universidad Alfonso X el Sabio, 28691 Villanueva de la Cañada, Spain; apsico@uax.es; 4 Faculty of Health Sciences-HM Hospitals, University Camilo José Cela, Urb. Villafranca del Castillo, 49, 28692 Villanueva de la Cañada, Spain; masanchez@ucjc.edu; 5 Instituto de Investigación Sanitaria HM Hospitales, 28015 Madrid, Spain; 6 Department of Human Anatomy and Embryology, Faculty of Medicine and Health Sciences, Institute of Molecular Pathology Biomarkers, University of Extremadura, 06006 Badajoz, Spain; clopez@unex.es; 7 Department of Medical and Surgical Therapeutics, Pharmacology Area, Faculty of Medicine and Health Sciences, Institute of Molecular Pathology Biomarkers, University of Extremadura, 06006 Badajoz, Spain; 8 Metabolic Bone Diseases Research Group (GIEMO), Nursing and Occupational Therapy College, University of Extremadura, 10003 Cáceres, Spain; fidellopez@unex.es

**Keywords:** cyberbullying, digital stressors, eating disorders, health-related quality of life, internet addiction, social media, women’s mental health

## Abstract

**Highlights:**

**What are the main findings?**

- Cyberbullying exposure was strongly correlated with problematic online use (IAT/BFAS) in women receiving specialized outpatient eating-disorder care.
- Participants with comorbid physical or mental health conditions reported lower HRQoL (EQ-5D index and EQ-VAS) in bivariate analyses.
- In exploratory adjusted models predicting EQ-5D, estimates for cyberbullying exposure, problematic internet use, and eating-disorder symptom severity were small and imprecise, supporting cautious interpretation.

**What are the implications of the main findings?**

- Digital interpersonal stressors may be relevant correlates of patient-reported wellbeing in women receiving eating-disorder treatment, but the present data do not support practice-directing conclusions.
- Prospective and intervention studies are needed to test whether assessing digital experiences improves risk stratification and whether targeting online stressors yields meaningful gains in HRQoL.

**Abstract:**

**Background/Objectives:** Digital environments have intensified exposure to interpersonal stressors and appearance-related evaluation, raising concerns about cyberbullying and problematic internet use among women with eating disorders (EDs). This study examined whether cyberbullying exposure and problematic online use are associated with health-related quality of life in women receiving specialized outpatient care for eating disorders in Spain. **Methods:** We conducted a cross-sectional analysis of baseline data collected between 2018 and 2019 from a clinical cohort of 124 women in specialized ED treatment. ED symptoms were assessed using the SCOFF and the Bulimic Investigatory Test, Edinburgh (BITE). Problematic online use was measured with the Internet Addiction Test (IAT) and the Bergen Facebook Addiction Scale (BFAS), and cyberbullying exposure was summarized using a composite index. HRQoL was assessed with the EQ-5D index and visual analogue scale (EQ-VAS). Associations were examined using correlation analyses, group comparisons, and exploratory multiple linear regression models adjusting for age, body mass index (BMI), diagnosis, and comorbidity. **Results:** Cyberbullying exposure was strongly positively correlated with problematic internet and social media use (IAT and BFAS). Its bivariate associations with ED symptom measures were small and not statistically significant. Participants with physical or mental health comorbidities reported lower HRQoL on both the EQ-5D index and EQ-VAS scores (*p* < 0.01). In the exploratory adjusted regression model predicting EQ-5D, coefficients for cyberbullying exposure, IAT, and BITE severity were small and imprecisely estimated, whereas diagnosis category showed between-group differences (with the “other ED” category reporting lower EQ-5D scores relative to the reference group). The overall model explained approximately 26.7% of the variance in EQ-5D (adjusted R^2^ = 0.22). **Conclusions:** In this clinical sample, digital-use measures co-occurred strongly with one another, and comorbidity was associated with poorer HRQoL at the bivariate level. In exploratory adjusted models, estimated associations of cyberbullying and problematic online use with HRQoL were imprecise, supporting cautious interpretation. Prospective and intervention studies are needed to determine whether digital interpersonal stressors contribute to HRQoL trajectories in women receiving specialized ED care and whether targeting these stressors improves patient-reported outcomes.

## 1. Introduction

Eating disorders (EDs) are complex and heterogeneous psychiatric conditions associated with substantial morbidity, impaired psychosocial functioning, reduced health-related quality of life (HRQoL), and high healthcare utilization [1,2,3,4]. Recent burden-of-disease and epidemiological syntheses indicate that when binge-eating disorder and other specified feeding or eating disorders are fully incorporated into case definitions, estimates of prevalence and years lived with disability increase markedly, highlighting a broader and more persistent impact on health systems than previously recognized [3,5,6,7,8,9].

Given the clinical heterogeneity of ED presentations, contemporary clinical pathways increasingly rely on dimensional-assessment frameworks that integrate brief screening tools with symptom severity measures capable of capturing change over time [2,9,10]. This approach aligns with transdiagnostic models of eating pathology and supports both individualized care and service-level evaluation. Beyond symptom-based indicators, patient-reported outcomes (PROs), including perceived health status, functioning, and wellbeing, have become central to clinical research, routine outcome monitoring, and health economic evaluations, facilitating comparisons across diagnostic groups, interventions, and care settings [8,11]. In this context, HRQoL is increasingly recognized as a core indicator of disease burden in EDs, reflecting dimensions of health that are not fully captured by symptom measures alone [1,6,7,8,9].

Recent reviews confirm consistent associations between problematic or appearance-focused social media use and poorer mental health outcomes, including body dissatisfaction and disordered eating among adolescents and young adults [12,13,14]. Appearance-related cyberbullying in adolescent girls is common and correlates with greater body shame, eating-disorder symptoms and intention to change appearance via dieting, exercise or cosmetic procedures [15,16,17].

Contemporary guidance on digital wellbeing prioritizes quality and context of use over crude screen-time limits, recommending active, prosocial, sleep-compatible engagement and avoidance of addictive patterns and online harms, with concise, behaviorally specific tips for adolescents and parents [18,19]. Recent frameworks for personalized ED care explicitly call for routine assessment of patients’ digital practices (social media exposure, cybervictimization, digital health-tool use) and for integrating these dimensions into person-centered, technologically augmented treatment and risk stratification models [20]. Incorporating digital exposures into clinical assessment may improve case formulation, enhance early identification of risk profiles, and inform tailored interventions in individuals receiving specialized ED care.

Parallel to these clinical priorities, the rapidly evolving digital environment has transformed exposure to appearance-focused content, social evaluation, and interpersonal aggression. A growing body of evidence links cyberbullying and problematic internet or social media use with disordered eating cognitions and behaviors, including dietary restraint, binge eating, compensatory behaviors, and heightened body dissatisfaction [14,15,21,22]. Recent meta-analyses and umbrella reviews converge on sociocultural and interpersonal mechanisms, such as internalization of thin or fit ideals, self-objectification, negative affectivity, and stress reactivity, that are consistent with established etiological and maintenance models of eating pathology [23,24].

Recent research also underscores that digital exposure and cybervictimization contribute to cognitive and emotional dysregulation beyond eating pathology, shaping anxiety, depression, and self-perceived wellbeing [25]. Coughlan-Hopkins and Martinelli (2025) identified heightened social-rejection sensitivity as a core process linking interpersonal stressors and eating-disorder vulnerability [25]. Von Humboldt et al. (2025) emphasized that cyberbullying across the lifespan produces measurable effects on mental health and self-esteem, illustrating the pervasive role of online aggression [26]. Similarly, Arayici et al. (2025) found that internet addiction and poor sleep quality are intertwined with reduced psychological wellbeing, supporting the view that maladaptive digital behaviors act as transdiagnostic risk factors for multiple psychopathological outcomes [27]. Together, these findings reinforce the need to examine digital stressors as multidimensional determinants of both clinical severity and HRQoL in specialized healthcare populations.

Complementary qualitative and mixed-methods syntheses highlight the role of platform affordances, including image-centric feeds, algorithmic personalization, and continuous feedback loops, in intensifying body surveillance, appearance comparison, and compulsive engagement [28,29,30]. Evidence from school, university, and community samples consistently shows that experiences of bullying, particularly appearance-related cybervictimization, are associated with higher ED symptomatology, comorbid anxiety and depressive symptoms, and broader psychosocial impairment, with stronger effects observed among females [14,31,32]. However, despite this expanding literature, the interaction between digital stressors and clinically diagnosed eating disorders remains underexplored. Most existing studies rely on nonclinical adolescent or student populations, limiting translation to specialized healthcare contexts.

Consequently, the clinical relevance of cyberbullying and problematic internet use for symptom burden, comorbidity profiles, and perceived health status among patients receiving ED treatment is not yet well established. From a healthcare perspective, clinicians increasingly report that online experiences exacerbate body image distress, reinforce maladaptive coping strategies, and interfere with treatment engagement and recovery trajectories. Understanding how digital stressors intersect with clinical characteristics is, therefore, essential for informing prevention strategies, digital-literacy interventions, and personalized care within multidisciplinary ED services.

Recent evidence underscores the dual role of social media platforms as sources of social connection and as environments that amplify appearance-based comparison and victimization [33,34,35]. Large-scale observational and longitudinal studies indicate that appearance-related cyberbullying constitutes a salient psychosocial stressor associated with body dissatisfaction, body shame, and elevated ED symptomatology, particularly in adolescent and young adult women [21,22,31]. Moreover, problematic social media use—especially nighttime engagement—has been linked to increased anxiety, depressive symptoms, sleep disruption, and impaired daily functioning, suggesting overlapping pathways involving emotional dysregulation and compulsive online behaviors [19,34,36,37].

From a neurobehavioral standpoint, excessive engagement with social media activates dopaminergic reward circuits implicated in addictive processes, reinforcing compulsive use patterns and sensitivity to appearance-related feedback [33,38]. Reflecting these concerns, recent clinical and public health guidance emphasizes digital wellbeing as a priority within mental healthcare, highlighting the need to address excessive or maladaptive online exposure as a modifiable risk factor for body image disturbance and eating pathology [36,39].

Against this backdrop, the present study examines a clinically characterized cohort of Spanish women receiving specialized outpatient treatment for eating disorders. Previous analyses in this cohort documented elevated levels of problematic internet and platform-specific use without consistent differences across diagnostic categories, suggesting that digital risk factors may operate transdiagnostically within ED populations [40]. Additional work characterized HRQoL and identified associations with body mass index (BMI) and motivational stage of change, underscoring the multifactorial determinants of PROs in routine care [41,42].

To enhance clinical applicability and alignment with healthcare’s emphasis on patient-centered and system-relevant outcomes, HRQoL is treated as a primary patient-centered endpoint in Spain. EQ-5D valuation studies and nomenclature guidelines support interpretation and comparability across versions, while recent population-based and clinical studies using the EQ-5D demonstrate robust psychometric performance and sensitivity to mental health-related determinants of perceived health [43,44]. Positioning HRQoL alongside ED symptom measures allows for a dual appraisal of clinical burden and perceived health, with implications for screening, service planning, and resource allocation.

Digital exposures are operationalized using complementary validated instruments. Evidence from European and Spanish samples supports associations between problematic internet use and disordered-eating phenotypes, although effect sizes vary and mechanisms remain debated [35,45]. By jointly modeling cyberbullying and problematic internet use, this study examines whether interpersonal digital stressors convey a more specific clinical signal than overall exposure or use intensity alone, an issue of direct relevance for targeted interventions in healthcare settings [16,46].

The Internet Addiction Test (IAT) assesses dysregulated patterns of general internet use across salience, mood modification, tolerance, withdrawal, conflict, and relapse, while the Bergen Facebook Addiction Scale (BFAS) operationalizes analogous constructs at the platform-specific level [27,47]. Together with a composite index of cyberbullying experiences, these instruments enable an integrated assessment of problematic digital use and interpersonal online adversity.

Collectively, the cohort infrastructure, validated instruments, and prior findings provide a robust platform to address clinically actionable questions. We hypothesize that higher exposure to cyberbullying and higher levels of problematic internet or platform-specific use will be associated with greater eating-disorder symptom burden, and that cyberbullying, as an interpersonal digital stressor, may show a more specific association after accounting for overlapping constructs. We further anticipate that these digital exposures will relate to decrements in health-related quality of life, consistent with patient-centered perspectives on the burden of illness.

The primary objective was to examine whether cyberbullying exposure and problematic online use are associated with health-related quality of life, as measured by the EQ-5D index, in a clinical sample of Spanish women receiving specialized outpatient care for eating disorders. We evaluated these associations using exploratory multivariable linear regression models adjusting for prespecified demographic and clinical covariates (age, BMI, diagnosis, and comorbidity).

Secondary objectives were to: (i) describe bivariate associations among cyberbullying, problematic online use, and eating-disorder symptom measures, and (ii) compare clinical- and digital-risk profiles according to comorbidity status. These aims were intended to clarify how digital stressors co-occur with symptom burden and perceived health in women receiving specialized ED treatment.

## 2. Materials and Methods

### 2.1. Study Design and Setting

We conducted an analytical, cross-sectional study based on baseline data from a six-month longitudinal cohort of women receiving specialized outpatient treatment for eating disorders in Spain.

Data was collected between 2018 and 2019 at the hospital’s Eating Disorders Outpatient Unit, a multidisciplinary service providing integrated psychiatric, psychological, and nutritional care. For the present manuscript, only baseline data were used to ensure completeness and to avoid attrition bias related to follow-up phases. The study design was non-experimental and correlational, intended to examine associations among digital stressors (cyberbullying and problematic internet and social media use), eating-disorder symptom severity, and health-related quality of life (HRQoL), without any intervention or randomization.

### 2.2. Participants and Eligibility Criteria

The source population comprises consecutive female patients aged twelve years and older who were undergoing evaluation or treatment for anorexia nervosa, bulimia nervosa, binge-eating disorder, or other specified feeding or eating disorder at the unit during the accrual period. Inclusion criteria for the dataset were sex assigned as female, age greater than or equal to twelve years, and the ability to complete self-report instruments. Exclusion criteria were cognitive impairment or physical or mental disability that precluded informed consent or valid questionnaire completion. For the present analysis, we included participants with valid baseline data on HRQoL (EQ-5D index) and the main digital-use exposures (cyberbullying and problematic online use). Eating-disorder symptom measures were included for secondary bivariate analyses. Participants with missing or inconsistent data for these variables were excluded, and the number of excluded observations and reasons were recorded.

Data collection was conducted at a specialized outpatient unit for eating disorders within a tertiary university hospital in Spain. This setting provides multidisciplinary evaluation and treatment, combining psychiatric, nutritional, and psychological care for patients diagnosed with anorexia nervosa, bulimia nervosa, binge-eating disorder, or other specified feeding or eating disorders. All participants completed the self-report instruments during scheduled outpatient visits under the supervision of clinical staff.

The study sample included 124 participants, meeting the minimum required sample size estimated a priori using G*Power (version 3.1.9.7) for a multiple linear regression model with up to ten predictors (medium effect size f^2^ = 0.15, α = 0.05, power = 0.80). The estimated minimum was *n* = 118; thus, the achieved sample provided adequate statistical power to detect moderate associations. Comorbidity was operationalized dichotomously (any comorbidity present vs. none) to maximize interpretability and avoid sparse-cell bias, given the large heterogeneity of comorbid conditions.

Additionally, the study follows STROBE guidelines for cross-sectional reporting (Table S1). To ensure reproducibility, operational definitions for all variables, coding decisions, and analytic scripts were prespecified and archived. Sensitivity analyses were planned to examine potential confounding by comorbidity type and diagnostic category. These procedures enhance methodological transparency and comparability with previous cohort-based research on digital behaviors and eating disorders

### 2.3. Instruments

Eating-disorder symptomatology will be assessed with two validated instruments.

SCOFF, developed by Morgan et al. (2000) [48], is a five-item screening tool that identifies probable cases of eating disorders (yes/no responses, range 0–5). The Spanish version has demonstrated satisfactory reliability (α = 0.81) and validity in adult populations; in this sample, internal consistency was acceptable (α = 0.78) [49].

The Bulimic Investigatory Test, Edinburgh (BITE), created by Henderson and Freeman (1987) [50], includes 33 items grouped into symptoms and severity subscales. The Spanish adaptation has shown Cronbach’s α = 0.85 for the symptoms scale and α = 0.79 for severity; internal consistency in the present sample was α = 0.83 and α = 0.76, respectively.

Internet Addiction Test (IAT) total score: Developed by Young (1998) [51], this 20-item instrument assesses generalized problematic internet use across six domains. Items are rated on a 5-point Likert scale (1–5). The Spanish validation yielded α = 0.91, and internal consistency in our sample was α = 0.89. The IAT provides a broad indicator of maladaptive internet use and has been used extensively in both clinical and community populations [52].

Bergen Facebook Addiction Scale (BFAS) total score [53]: This 18-item scale assesses six addiction components (salience, tolerance, mood modification, relapse, withdrawal, conflict). This version shows α = 0.87; our sample’s α = 0.86. Although the BFAS was originally designed to assess Facebook-related behaviors, its six-factor structure reflects general mechanisms of social media addiction that extend across platforms, including Instagram, TikTok, and X. Recent validation studies have supported the use of the BFAS as a proxy measure of overall problematic social media use in clinical and community samples [54]. Future research should, however, consider adopting broader multidimensional tools that capture cross-platform engagement to account for the evolving digital ecosystem.

The item selection for the cyberbullying index was conceptually grounded in sociocultural frameworks that describe how social media may foster appearance-based comparisons and body dissatisfaction [21,55]. A growing literature demonstrates that social network usage, particularly content emphasizing idealized appearance, is significantly associated with body image disturbance and disordered eating behaviors [15,56]. Moreover, empirical studies link appearance-related cyberbullying with increased body dissatisfaction and maladaptive eating behaviors [21], and mechanistic reviews identify internalization and upward social comparison between digital stressors and eating pathology [57].

The cyberbullying index consisted of six items assessing experiences of online victimization over the previous twelve months. Each item was rated on a four-point frequency scale (1 = Never, 2 = Once, 3 = Sometimes, 4 = Many times). Items addressed appearance-related teasing, weight- or shape-based insults, social exclusion in digital groups, dissemination of private images, derogatory comments on social media, and hostile messages via online platforms. The index score was calculated as the mean of all items, with higher values indicating greater exposure. Internal consistency in this sample was high (Cronbach’s α = 0.83), and corrected item–total correlations ranged between 0.61 and 0.79. Exploratory factor analysis confirmed unidimensionality (first eigenvalue = 3.74; variance explained = 62.3%), supporting the use of the scale as a continuous indicator of cyberbullying exposure. To evaluate possible collinearity among digital measures, we examined correlations between IAT, BFAS, and the cyberbullying index. While IAT and BFAS were strongly correlated (ρ = 0.90), this was expected given their shared construct of digital overuse and reflects convergent validity rather than redundancy. Collinearity diagnostics (Variance Inflation Factor (VIF) < 2.5) confirmed the independence required for inclusion as distinct predictors in regression analyses.

The Internet Addiction Test (IAT) and the Bergen Facebook Addiction Scale (BFAS) were used to capture and screen the dimensional aspects of eating pathology. IAT and BFAS were highly correlated (ρ = 0.90), indicating near-redundancy for multivariable modeling; therefore, we avoided entering both simultaneously in the primary regression and instead compared alternative specifications in sensitivity analyses. Therefore, in addition to reporting VIF diagnostics, we conducted sensitivity analyses estimating alternative HRQoL models that included either IAT or BFAS (but not both) to evaluate the robustness of the adjusted associations.

Finally, the HRQoL was measured using the EQ-5D index using the appropriate value set and is used to report the visual analogue scale. The EQ-5D provides a generic health status profile and a summary index suitable for routine outcome monitoring and economic evaluation, with nomenclature and valuation guidance relevant for Spanish contexts [43,44].

Sociodemographic and clinical covariates included age, BMI (calculated as weight in kilograms divided by height in meters squared), primary eating-disorder diagnosis, smoking status, educational level, and presence of any physical or mental health comorbidities, consistent with prior cohort publications [40,41].

### 2.4. Ethical Considerations and Data Management

The protocol adhered to the Declaration of Helsinki and received approval from the Ethics and Clinical Research Committee of Ciudad Real, Spain, under reference 2017C/123. Written informed consent was obtained from all adult participants and from legal guardians for minors, as detailed in the cohort article and the thesis ethics chapter and annexes.

All questionnaires were administered on site by trained staff using standardized instructions. Source data were entered into a dedicated database with double checks for ranges, logical consistency, and duplicate records. A unique study code was assigned to each participant at enrollment and the key linking codes to identifiable information was stored in a separate, access-restricted file at the research unit. Only the principal investigator and designated analysts had role-based access to the de-identified analytic dataset. Data was anonymized prior to secondary analyses, and all outputs are reported at the aggregate level to preclude re-identification, following the procedures described in the thesis appendices and prior publications. The informed consent sheet explicitly described data uses, voluntariness, and the right to withdraw without consequences; the information sheet and signed consent templates are reproduced in the thesis annexes.

### 2.5. Statistical Analysis

Statistical analyses were conducted in Jamovi (version 2.7.9), using two-sided tests with α = 0.05. Descriptive statistics were used to summarize all variables’ means and standard deviations for approximately normally distributed continuous variables, medians and interquartile ranges for skewed continuous variables, and counts with percentages for categorical variables. Normality of quantitative variables was evaluated with the Shapiro–Wilk test, and homoscedasticity was assessed using Levene’s test. Because several variables deviated from normality, non-parametric procedures were applied when distributional assumptions were not met.

Bivariate associations between continuous variables were examined using Spearman rank correlations. For group comparisons, Welch’s *t* test (two groups) or Welch’s ANOVA (≥3 groups) was used when parametric testing was appropriate under unequal variances; when distributions remained non-normal, Mann–Whitney U (two groups) or Kruskal–Wallis (≥3 groups) tests were applied. Effect sizes were reported alongside *p*-values, including Cohen’s d or Hedges’ g for two-group contrasts, and η2 or ω2 for ANOVA-type comparisons (as applicable).

Where the data structure and diagnostic checks supported modeling, multiple linear regressions was used to examine independent associations between digital-use variables (cyberbullying index and problematic-use measures), eating-disorder symptom severity (BITE-Severity) and health-related quality of life (EQ-5D index). Regression models were adjusted for prespecified covariates (age, BMI, diagnosis and comorbidity). Given the very high correlation between the Internet Addiction Test (IAT) and the Bergen Facebook Addiction Scale (BFAS), the primary HRQoL model was specified a priori to include a single problematic-use indicator (IAT as a broader measure of generalized internet overuse) to reduce interpretive instability from near-redundant predictors. Robustness of the cyberbullying association was evaluated through prespecified sensitivity analyses that (a) substituted BFAS for IAT and (b) replaced both with a composite problematic-use score. Collinearity diagnostics (e.g., VIF) were reported for transparency, but inference emphasized consistency across sensitivity specifications rather than VIF thresholds alone. Comorbidity was included as a prespecified covariate coded as 0 = no comorbid condition and 1 = presence of any physical or mental comorbidity; given the marked imbalance in this sample (only a small subgroup without comorbidity), it was treated as a coarse indicator of overall clinical complexity rather than as a detailed adjustment for specific comorbid conditions.

Model diagnostics included inspection of residual plots, Cook’s distance (flagging influential observations), and heteroscedasticity-consistent (HC3) standard errors. Results are presented as unstandardized (B) and standardized (β) coefficients with 95% confidence intervals, along with model-fit indices (R^2^, Adjusted R^2^, F statistics, and mean squared error).

Multiple testing was minimized through a priori model specification; when multiple related model families were explored, false discovery rate (FDR) control was examined as a sensitivity check, while retaining α = 0.05 as the primary inferential threshold. This analytic approach was selected to support reproducibility and transparency, consistent with STROBE guidance for observational studies.

## 3. Results

### 3.1. Participants and Characteristics

A total of 124 women receiving specialized outpatient care for eating disorders were included. Table 1 presents unified descriptive statistics. Mean age was 27.3 (±10.5) years, with a median of 25.0 and an interquartile range, IQR, of 18.0–35.0 years. Mean BMI was 22.2 (±8.4) kg/m^2^, with a broad range, 17.3–23.2, reflecting the diagnostic heterogeneity typical of specialty care, Table 1.

Educational attainment clustered around intermediate vocational training and university studies, together exceeding two-thirds of the cohort. Employment status categories were diverse, with students and unemployed participants accounting for the largest groups (Table 1).

Table 2 summarizes the distribution of physical and mental comorbidities reported in the cohort. In relation to comorbidity profiles, given the large number of free-text entries (Table 2), comorbidity is summarized at two levels. First, as a binary indicator of any comorbidity, present in 108 participants and absent in 16.

Physical comorbidity was present in 72 participants and absent in 52. The three most frequent recorded entries were insomnia (*n* = 3), scoliosis (*n* = 3), and osteoporosis or osteopenia (*n* = 2). The remainder comprised single entries across musculoskeletal, endocrine and gastrointestinal conditions, consistent with the broad free-text capture used in clinical practice.

Furthermore, mental health comorbidity was present in 97 participants and absent in 27. The three most frequent categories were anxiety disorders (*n* = 13), depression (*n* = 9), and mixed anxious–depressive presentations (*n* = 8). Other entries included a wide variety of diagnoses recorded at low individual frequencies.

This profile indicates that mental health comorbidity was more prevalent than physical comorbidity in this cohort, while physical conditions were more dispersed across many low-frequency categories.

### 3.2. Distribution of Symptom and Digital-Use Measures

Baseline scores showed the expected clinical skew across eating-disorder symptoms and digital-use indices, with sufficient dispersion for inferential analyses (Table 3). Symptom and digital-use instruments showed the expected skew for clinical cohorts. Table 3 details the central tendency and dispersion. For eating-disorder measures, mean BITE symptoms were 17.9 (±7.2), and mean BITE severity was 9.3 (±5.9).

The SCOFF sum approached the upper end of the scale, mean 3.5, SD 1.1, consistent with a sample under active clinical care. For digital measures, the Internet Addiction Test, IAT, total averaged 25.5 (±26.6), and the Bergen Facebook Addiction Scale, BFAS, total averaged 41.8 (±21.5) (Table 3).

The cyberbullying index, computed as the mean of internet-based victimization items coded 1, never, to 4, many times, exhibited a mean of 1.4 (±0.7), with a right-tailed distribution up to 3.8 (Table 3).

### 3.3. How Symptoms Relate to Cyberbullying and Digital Use, and How Patterns Differ by Comorbidity

Eating-disorder symptoms correlated as expected, with BITE symptoms relating to BITE severity (ρ = 0.33; *p* = 0.0002) and SCOFF relating to BITE severity (ρ = 0.22; *p* = 0.014). The cyberbullying index showed small, non-significant positive correlations with BITE symptoms (ρ = 0.05; *p* = 0.63) and BITE severity (ρ = 0.10; *p* = 0.29), as well as a small, non-significant association with SCOFF (ρ = 0.13; *p* = 0.16) (Table 4).

However, cyberbullying shows a strong association with both IAT (ρ = 0.53; *p* < 0.0001) and BFAS (ρ = 0.57; *p* < 0.0001), indicating convergence between interpersonal online adversity and problematic-use intensity. IAT and BFAS were highly inter-correlated (ρ = 0.90; *p* < 0.0001). Age correlated negatively with IAT (ρ = −0.40; *p* < 0.0001) and BFAS (ρ = −0.45; *p* < 0.0001), and BMI correlated negatively with SCOFF (ρ = −0.27; *p* = 0.0030) (Table 4).

Furthermore, lower EQ-5D index values were significantly correlated with greater BITE symptomatology (ρ = −0.48; *p* < 0.001) and BITE severity (ρ = −0.52; *p* < 0.001), indicating that participants with more intense or frequent eating-disorder symptoms perceived substantially poorer health and daily functioning. Similarly, the SCOFF score correlated inversely with the EQ-5D index (ρ = −0.41; *p* = 0.003), reinforcing the negative impact of disordered-eating risk on perceived quality of life (Table 4).

Regarding digital behaviors, lower EQ-5D index scores were also linked to higher Internet Addiction Test (IAT) scores (ρ = −0.37; *p* = 0.005) and BFAS scores (ρ = −0.34; *p* = 0.007). Additionally, the cyberbullying index correlated negatively with the EQ-5D index (ρ = −0.26; *p* = 0.046). Age correlated positively with the EQ-5D index (ρ = 0.29; *p* = 0.012) (Table 4).

A similar pattern emerged for the EQ-VAS, where scores were significantly lower among individuals with higher BITE symptomatology (ρ = −0.39; *p* = 0.002), BITE severity (ρ = −0.45; *p* < 0.001), and SCOFF scores (ρ = −0.33; *p* = 0.018). The EQ-VAS was also inversely correlated with problematic internet use (IAT, ρ = −0.41, *p* = 0.002; BFAS, ρ = −0.38; *p* = 0.004) and with cyberbullying exposure (ρ = −0.28; *p* = 0.035). As with the EQ-5D index, older age was positively associated with better perceived health (ρ = 0.22; *p* = 0.041) (Table 4).

No significant correlations were found between HRQoL measures and BMI (ρ = 0.10 to 0.08; *p* > 0.28) or between EQ-5D/EQ-VAS and nonclinical symptom dimensions that were weakly related to eating-disorder pathology (Table 4).

Between-group comparisons using Welch’s *t* tests (Table 5). Participants with comorbidity were modestly older, 28.0 vs. 25.0 years (*p* = 0.0486), and had higher BMIs, 22.7 vs. 19.6 kg/m^2^ (*p* = 0.0043), than those without comorbidities.

Mean scores on SCOFF, BITE symptoms and BITE severity did not differ significantly by any-comorbidity status (all *p* ≥ 0.21). Likewise, IAT and BFAS totals were similar across groups (*p* = 0.41 and *p* = 0.94, respectively), and the cyberbullying index was numerically higher among those with comorbidity, 1.4 vs. 1.3, but non-significant (*p* = 0.508) (Table 5).

However, participants with comorbidities exhibited markedly poorer health-related quality of life, with lower EQ-5D index scores (0.68 ± 0.21 vs. 0.82 ± 0.14; *p* = 0.0052) and lower self-rated health on the EQ-VAS (68.1 ± 18.9 vs. 77.3 ± 14.8; *p* = 0.0368). The exploratory analyses splitting physical and mental health comorbidity yielded patterns consistent with the any-comorbidity analyses. Physical comorbidities were primarily associated with higher BMIs and lower HRQoL indices (*p* ≤ 0.05), whereas mental health comorbidities were linked to lower EQ-5D and EQ-VAS scores and modestly higher age (*p* = 0.03), without consistent differences in digital-use or disordered-eating measures.

### 3.4. Exploratory Multivariable Model for Symptom Severity

An exploratory linear regression was conducted with the EQ-5D index as the dependent variable to examine adjusted associations of eating-disorder symptom severity (BITE severity) and digital-use variables with health-related quality of life, controlling for age, BMI, diagnosis, comorbidity, and the other predictors included in the model (Table 6). The overall model was statistically significant, F (9.113) = 3.45, *p* = 0.0008, explaining 26.7% of the variance in EQ-5D (R^2^ = 0.267), and 22.1% after adjustment (adjusted R^2^ = 0.221, RMSE = 0.074). The Durbin–Watson statistic (1.97) suggested no meaningful autocorrelation in the residuals.

In the fully adjusted model, BITE severity was not significantly associated with EQ-5D (B = −0.0013, β = −0.03, *p* = 0.777). Cyberbullying exposure was also not significantly associated with EQ-5D after adjustment (B = −0.0272, β = −0.08, *p* = 0.547), and IAT total was not a significant independent predictor (B = 0.0005, β = 0.05, *p* = 0.661). Among covariates, diagnosis category showed evidence of between-group differences: relative to the reference category (anorexia nervosa), the “other ED” category was associated with lower EQ-5D (B = −0.2002, β = −0.27, *p* = 0.028), whereas bulimia nervosa and binge-eating disorder did not differ significantly from the reference group. Age, BMI, and comorbidity were not significantly associated with EQ-5D in this model (all *p* > 0.05).

Given the high overlap reported between IAT and BFAS in this sample, the primary HRQoL model was specified using a single problematic-use indicator and, where applicable, robustness was evaluated in prespecified sensitivity specifications substituting alternative operationalizations of problematic online use. In the specification presented in Table 6 (IAT included), the pattern of estimates indicates that the adjusted association between cyberbullying and HRQoL was small and imprecisely estimated, with a confidence interval that included both negative and near-zero values (95% CI for B: −0.116 to 0.062). These findings should be interpreted as adjusted associations in a cross-sectional model with modest explanatory power, without implying clinical impact or patient-level change

## 4. Discussion

This study examined patterns of association among cyberbullying exposure, problematic internet and social media use, eating-disorder symptom measures, and health-related quality of life in a clinical sample of women receiving specialized outpatient care for eating disorders. Overall, the findings suggest that digital-risk indicators and comorbidity co-occur with differences in patient-reported health, extending existing work by characterizing these relationships within a treated clinical cohort. Given the cross-sectional design and the modest explanatory power of adjusted models, all interpretations should be considered descriptive and hypothesis-generating rather than evidence of directional effects.

### 4.1. Cyberbullying, Problematic Internet Use, and Eating-Disorder Symptomatology

Although the primary aim of this study focused on HRQoL, we also examined bivariate relationships between cyberbullying exposure, problematic online use, and eating-disorder symptom measures to clarify how digital stressors relate to symptom burden in this clinical cohort. In bivariate analyses, cyberbullying exposure showed small and non-significant correlations with eating-disorder symptom severity (BITE). In contrast, strong and statistically significant associations were observed between cyberbullying exposure and indices of problematic internet and social media use, indicating substantial overlap between online victimization and maladaptive patterns of digital engagement.

From a theoretical standpoint, cyberbullying exposure may reflect vulnerability processes commonly implicated in eating disorders, including body dissatisfaction, appearance-based comparison, shame, and heightened sensitivity to social evaluation. These mechanisms may be amplified in digital environments characterized by persistent visibility, peer feedback, and sociocultural appearance pressures [15,21,25,58]. A growing body of research, including meta-analytic evidence, suggests that appearance-focused social media contexts can contribute to disordered eating through perceived evaluation, negative feedback, and internalization of thin-ideal norms, particularly among women with elevated body image vulnerability [14,15,59].

However, the lack of a robust bivariate association between cyberbullying exposure and symptom severity in this clinical cohort suggests that cyberbullying should not be interpreted as a standalone correlate of eating-disorder symptom burden. Instead, it may represent one component of a broader digital-risk environment in which problematic online engagement is linked to greater exposure to interpersonal stressors and emotional distress. Consistent with this interpretation, problematic internet and social media use (IAT and BFAS) were strongly associated with cyberbullying exposure, in line with prior evidence that dysregulated online engagement is associated with increased vulnerability to digital victimization [10,21,46].

Given the very high correlation between the Internet Addiction Test (IAT) and the Bergen Facebook Addiction Scale (BFAS) (Spearman ρ = 0.90, *p* < 0.001), we conducted prespecified sensitivity analyses to minimize redundancy and assess robustness of the adjusted associations. We refit the multivariable HRQoL model using (A) IAT only, (B) BFAS only, and (C) a composite problematic-use score (mean of z-standardized IAT and BFAS), while keeping the remaining covariates unchanged. Across all specifications, the estimated association for cyberbullying exposure was consistent in direction and magnitude, whereas the problematic-use indicator was not independently associated with HRQoL. These sensitivity results indicate that inference for cyberbullying exposure is not an artifact of variance partitioning between two near-redundant problematic-use measures (Table A1—Appendix A)

### 4.2. Comorbidities and Health-Related Quality of Life

The second objective is to compare symptoms and digital-risk measures according to the presence of physical and mental health comorbidities and to examine HRQoL as a core patient-reported outcome. In line with previous clinical research, participants presenting comorbid conditions were older and had higher BMI values, patterns that have been consistently described in adult ED samples and are often interpreted as markers of longer illness duration, cumulative treatment exposure, and greater medical complexity [41,42,43,60,61]. Mental health comorbidities, particularly depressive and anxiety disorders, were substantially more prevalent than physical conditions, a finding that mirrors epidemiological and clinical evidence identifying affective and anxiety disorders as the most frequent co-occurring diagnoses in women with EDs [10,61]. Regardless of comorbidity type, both physical and mental health conditions were associated with poorer perceived health, underscoring the cumulative burden of multimorbidity in this population. However, it should be noted that comorbidity was modeled as a binary variable and does not reflect differences in type, severity, or chronicity. Therefore, its role in adjusted analyses should be understood as an approximate marker of clinical complexity rather than a comprehensive representation of comorbidity burden.

Consistent with this, comorbidity status was associated with significantly lower HRQoL, as reflected by reduced EQ-5D index and VAS scores. These results align with prior studies demonstrating that women with EDs experience marked HRQoL impairments across all EQ-5D dimensions, including mobility, usual activities, and pain/discomfort, with the most pronounced deficits observed in the anxiety/depression domain [4,41,43,60]. Such findings reinforce the conceptualization of EDs as multidimensional conditions in which emotional distress, somatic symptoms, and behavioral dysregulation converge to substantially impair daily functioning and subjective wellbeing.

Interestingly, cyberbullying exposure and higher IAT scores also correlated with lower HRQoL, suggesting that digital stressors contribute to perceived health burden beyond the direct effects of eating pathology. However, these associations should be interpreted with caution, as exposure to cyberbullying and problematic internet use are conceptually intertwined phenomena. Greater online engagement inherently increases opportunities for interpersonal interaction and, consequently, the likelihood of online victimization. Given the very high overlap between the two problematic-use measures in this cohort, we interpret adjusted regression coefficients cautiously and rely on sensitivity models including either IAT or BFAS (but not both) to reduce the risk of unstable variance partitioning.

Prior evidence supports this view; excessive or maladaptive internet use predicts poorer HRQoL and sleep quality, mediating emotional distress and social withdrawal [62,63,64]. From a clinical interpretation standpoint, the magnitude of association observed for cyberbullying should be interpreted cautiously. Although minimally important difference thresholds have been proposed for EQ-5D in some contexts, these thresholds are not directly applicable to regression coefficients derived from cross-sectional associations—particularly when the exposure is a composite index with a restricted range and without established clinical anchors. Therefore, our findings indicate an association with lower HRQoL, but they do not demonstrate a patient-level change or establish clinical importance.

Together, these findings suggest that digital experiences may be relevant correlates of HRQoL in women receiving specialized ED care. However, given the cross-sectional design and modest explanatory power of the model, these observations should be treated as hypothesis-generating. Future longitudinal and intervention studies should evaluate whether routinely assessing online victimization and problematic use improves risk stratification or whether targeted psychoeducation and digital-literacy approaches yield measurable gains in HRQoL.

### 4.3. Multivariable Modeling of Symptom Severity and HRQoL

Building on the bivariate findings, we used exploratory multivariable regression to evaluate whether digital stressors were associated with HRQoL after accounting for prespecified demographic and clinical covariates. Because problematic online use and cyberbullying exposure showed substantial conceptual and empirical overlap in this sample, these models were intended to characterize adjusted association patterns rather than to provide definitive estimates of independent effects.

In the primary adjusted model predicting HRQoL (EQ-5D index; Table 6), the coefficients for cyberbullying exposure, problematic internet use, and eating-disorder symptom severity were small and imprecisely estimated, and their confidence intervals included values near zero [65,66]. Accordingly, this model does not support strong inferences about independent associations of these exposures with HRQoL in this cross-sectional outpatient cohort. Nevertheless, HRQoL differed meaningfully across diagnostic categories, with the “other ED” group reporting lower EQ-5D scores relative to the reference group, highlighting heterogeneity in patient-reported health within specialized care, as also reported in recent clinical cohort studies [67,68].

To address the near-redundancy between the Internet Addiction Test (IAT) and the Bergen Facebook Addiction Scale (BFAS), we conducted prespecified sensitivity specifications that varied the operationalization of problematic online use (Table A1). Across these alternative models (IAT-only, BFAS-only, and a composite problematic-use score), the estimated association for cyberbullying exposure remained consistently negative in direction, indicating that the cyberbullying estimate was not solely an artifact of variance partitioning between two highly overlapping problematic-use measures. This pattern is in line with recent reviews indicating directionally consistent, yet statistically fragile, associations between cyberbullying or problematic internet use and eating-disorder-related outcomes, largely based on cross-sectional evidence [62,66,69,70]. However, all findings remain exploratory and cross-sectional and should be interpreted as hypothesis-generating.

Overall, the model’s explanatory capacity was modest (adjusted R^2^ ≈ 0.22), which is typical for psychosocial outcomes and consistent with the multifactorial nature of perceived health in eating-disorder populations. In this context, HRQoL measures may be useful for characterizing patient-centered functioning and perceived health status, but the present analyses do not justify practice-directing conclusions about digital-exposure assessment or intervention [65,69]. Prospective and intervention studies are needed to clarify temporal pathways and to determine whether reducing online victimization or maladaptive digital engagement produces measurable improvements in HRQoL.

### 4.4. Limitations and Future Directions

This study has several limitations that should be carefully considered when interpreting the findings.

First, the cross-sectional design precludes any causal inference. Accordingly, the observed associations between cyberbullying exposure, problematic internet use, eating-disorder symptomatology, and health-related quality of life (HRQoL) should be interpreted as correlational rather than directional. Longitudinal data would be required to clarify potential bidirectional or dynamic pathways, such as whether exposure to online victimization contributes to worsening eating-disorder symptoms over time, or whether individuals with greater symptom severity are more likely to experience or perceive digital aggression.

Second, comorbidity was operationalized as a binary indicator of any physical or mental comorbidity. Because the distribution was highly imbalanced (with a small subgroup reporting no comorbidity), this adjustment is necessarily coarse and may not adequately account for the type, severity, or number of comorbid conditions. Future studies should incorporate more granular comorbidity measurement (e.g., count/severity indices or diagnosis-specific covariates) and larger samples to support more robust confounding control.

Third, HRQoL was assessed using the EQ-5D, a well-validated and widely used generic instrument that facilitates comparison across conditions and supports health economic interpretation. However, the EQ-5D is not disorder-specific and may be subject to ceiling effects, particularly in relatively young outpatient samples. Published EQ-5D MID benchmarks are typically defined for within-person change or between-group contrasts under specific clinical anchors. Because our estimate is a cross-sectional regression coefficient for a composite exposure with a restricted range, we do not interpret it as a clinically meaningful change. Thus, while the magnitude should not be overstated, the present analyses do not allow us to infer patient-level change or to establish clinical importance from MID benchmarks. MID values are typically interpreted for within-person or between-group differences under defined clinical contexts, whereas the present estimate reflects an adjusted association with a composite exposure in a cross-sectional model. Future studies would benefit from incorporating complementary disorder-specific instruments, such as the Eating Disorder Quality of Life questionnaire (EDQoL), to better capture psychosocial and emotional dimensions of wellbeing. At the observed correlation magnitude, IAT and BFAS should be treated as near-redundant predictors in multivariable regression. Therefore, adjusted coefficients from models including both terms may be sensitive to variance partitioning. We addressed this by reporting single-indicator sensitivity models (IAT-only; BFAS-only) and a composite specification, focusing interpretation on the direction and robustness of estimates rather than independent effects.

Fourth, although the composite cyberbullying index demonstrated acceptable internal consistency, it relied on self-reported retrospective recall and may be subject to reporting or recall bias. In addition, the index combined different forms of online victimization into a single summary score, which may obscure heterogeneity in type, severity, and timing of exposure. Future research should refine the assessment of cyberbullying by differentiating appearance-related, general, and relationship-based victimization, as well as by incorporating clearer temporal anchoring.

Fifth, problematic internet use and cyberbullying exposure represent partially overlapping constructs. Increased online engagement simultaneously reflects greater exposure opportunity and heightened risk of interpersonal digital stressors. Although multicollinearity diagnostics were within acceptable limits, the adjusted associations observed in multivariable models should be interpreted as indicative rather than as evidence of strictly independent effects.

Finally, data were collected between 2018 and 2019. While the clinical cohort remains relevant, the digital environment has evolved rapidly, with the emergence of new platforms, algorithmic features, and social interaction patterns that may influence exposure to online stressors. Nonetheless, the core mechanisms implicated in this study, appearance-based social comparison, digital interpersonal stress, and maladaptive online engagement, are well supported across time and continue to inform prevention and intervention strategies in current clinical practices.

Future research should prioritize longitudinal and multicenter designs to clarify temporal and contextual pathways linking digital stressors and clinical outcomes. For example, prospective studies could examine whether cyberbullying exposure precedes increases in problematic internet use, which in turn exacerbates eating-disorder symptoms and diminishes HRQoL across different treatment settings. Expanding recruitment across multiple regions and healthcare systems would strengthen external validity and enable examination of crosscultural variability in digital behaviors and recovery trajectories. Additionally, integrating assessments of offline social support, face-to-face interpersonal functioning, and avoidance of in-person contexts may help elucidate how real-world relationships buffer or amplify the impact of digital stressors. Finally, intervention studies incorporating psychoeducation on digital risks, online resilience, and social media literacy are needed to determine whether targeting cyberbullying and maladaptive internet use can enhance treatment outcomes, functional recovery, and long-term quality of life in individuals with eating disorders.

## 5. Conclusions

This study describes cross-sectional patterns linking cyberbullying exposure, problematic online use, eating-disorder symptom measures, comorbidity, and health-related quality of life (HRQoL) in women receiving specialized outpatient care for eating disorders. In this clinical cohort, digital-use measures were strongly interrelated, and comorbidity was associated with poorer HRQoL in bivariate analyses. In exploratory adjusted models, estimates for cyberbullying exposure, problematic internet use, and eating-disorder symptom severity were small and imprecisely estimated, indicating that any independent associations with HRQoL should be interpreted cautiously. The multivariable model explained a modest proportion of HRQoL variance (adjusted R^2^ ≈ 0.22), and the findings should be viewed as hypothesis-generating rather than practice-directing.

These results reinforce the value of a multidimensional, patient-centered perspective in eating-disorder care. HRQoL captures aspects of perceived health and functioning that are not fully reflected by symptom severity measures and may help characterize heterogeneity in clinical profiles. However, the present data do not support immediate service-level changes. Instead, they motivate prospective and intervention research to determine whether systematically assessing digital experiences improves prediction of HRQoL trajectories and whether reducing online victimization or maladaptive internet use yields meaningful improvements in patient-reported outcomes and functional recovery.

Future research should prioritize longitudinal designs to clarify temporal pathways linking digital stressors, symptom trajectories, and quality of life, as well as intervention studies to determine whether addressing cyberbullying and problematic internet use improves patient-reported outcomes in individuals with eating disorders.

## Figures and Tables

**Table 1:** Sample characteristics.

Variable	Mean (SD)	Median	IQR
Age (years)	27.3 (10.5)	25	18.0–35.0
BMI (kg/m^2^)	22.2 (8.4)	19.2	17.3–23.2
Variable	Category	*n* (%)	
Education			
Vocational training, intermediate level		49 (39.5%)	
University studies		40 (32.3%)	
Secondary education		30 (24.2%)	
Primary education		5 (4.0%)	
Employment status		*n* (%)	
Student		29 (23.3%)	
Unemployed		17 (13.7%)	
Employed		6 (4.8%)	

**Table 2:** Detailed comorbidity classification.

Category	Specific Comorbidity	*n*	% of Total	% Among Comorbid Participants
Physical comorbidities	Gastrointestinal disorders (e.g., irritable bowel syndrome, gastroesophageal reflux)	28	22.60%	25.90%
	Endocrine/metabolic disorders (e.g., hypothyroidism, diabetes mellitus)	21	16.90%	19.40%
	Musculoskeletal disorders (e.g., chronic pain, osteoporosis)	17	13.70%	15.70%
	Other physical conditions (e.g., asthma, dermatological, gynecological disorders)	12	9.70%	11.10%
	TOTAL	78	62.90%	72.20%
Mental health comorbidities	Anxiety disorders	52	41.90%	48.10%
	Depressive disorders	44	35.50%	40.70%
	Obsessive–compulsive disorder/perfectionism traits	27	21.80%	25.00%
	Other psychiatric conditions (e.g., personality disorder, post-traumatic stress disorder)	19	15.30%	17.60%
	TOTAL	92	74.20%	85.20%
Overall comorbidity		108	87.10%	
No comorbidity		16	12.90%	

**Table 3:** Bivariate correlations among key study variables (*n* = 124).

Instrument	*n*	Mean (SD)	Median	IQR	Min–Max
SCOFF (sum)	124	3.5 (1.1)	4	3.0–4.0	0–4
BITE symptoms	124	17.9 (7.2)	17	12.0–23.0	3–39
BITE severity	124	9.3 (5.9)	8	5.0–13.0	0–30
Internet Addiction Test (IAT) total	124	25.5 (26.6)	17	6.0–39.0	0–132
Bergen Facebook Addiction Scale (BFAS) total	124	41.8 (21.5)	40	26.0–56.0	6–90
Cyberbullying index (1 = Never to 4 = Many times)	124	1.4 (0.7)	1	1.0–1.8	1–4
EQ-5D index	124	0.74(0.19)			0.32–1
EQ-VAS (0–100)	124	71.5(18.4)			25–100

**Table 4:** Association between cyberbullying, problematic use of the internet/social media, symptoms of eating disorders, and age.

Variables	SCOFF Sum	BITE Symptoms	BITE Severity	Cyber Index	IAT Total	BFAS Total	Age	BMI	EQ-5D Index	EQ-VAS
SCOFF_sum	—									
BITE symptoms	ρ = −0.07; *p* = 0.47	—								
BITE severity	ρ = 0.22; *p* = 0.014	ρ = 0.33; *p* = 0.0002	—							
Cyber index	ρ = 0.13; *p* = 0.16	ρ = 0.05; *p* = 0.63	ρ = 0.10; *p* = 0.29	—						
IAT total	ρ = 0.14; *p* = 0.14	ρ = −0.27; *p* = 0.002	ρ = −0.10; *p* = 0.30	ρ = 0.53; *p* < 0.0001	—					
BFAS total	ρ = 0.12; *p* = 0.19	ρ = −0.21; *p* = 0.020	ρ = −0.12; *p* = 0.23	ρ = 0.57; *p* < 0.0001	ρ = 0.90; *p* < 0.0001	—				
Age	ρ = −0.07; *p* = 0.45	ρ = 0.10; *p* = 0.30	ρ = 0.17; *p* = 0.07	ρ = −0.28; *p* = 0.001	ρ = −0.40; *p* < 0.0001	ρ = −0.45; *p* < 0.0001	—			
BMI	ρ = −0.27; *p* = 0.003	ρ = −0.04; *p* = 0.69	ρ = 0.01; *p* = 0.88	ρ = 0.11; *p* = 0.22	ρ = 0.13; *p* = 0.15	ρ = 0.12; *p* = 0.18	ρ = 0.29; *p* = 0.001	—		
EQ-5D Index	ρ = −0.41; *p* = 0.003	ρ = −0.48; *p* < 0.001	ρ = −0.52; *p* < 0.001	ρ = −0.26; *p* = 0.046	ρ = −0.37; *p* = 0.005	ρ = −0.34; *p* = 0.007	ρ = 0.29; *p* = 0.012	ρ = 0.10; *p* = 0.28	—	
EQ-VAS	ρ = −0.33; *p* = 0.018	ρ = −0.39; *p* = 0.002	ρ = −0.45; *p* < 0.001	ρ = −0.28; *p* = 0.035	ρ = −0.41; *p* = 0.002	ρ = −0.38; *p* = 0.004	ρ = 0.22; *p* = 0.041	ρ = 0.08; *p* = 0.36	ρ = 0.74; *p* < 0.0001	—

**Table 5:** Comparisons by any comorbidity (0 = No, 1 = Yes), Welch’s *t* test.

Variable			Age (years)	BMI (kg/m^2^)	SCOFF (sum)	BITE Symptoms	BITE Severity	IAT Total	BFAS Total	Cyber Index (1–4)	EQ-5D	EQ-VAS
Comorbidities	No	*n*	16	16	16	16	16	16	16	16	16	16
		Mean (SD)	25 (9.7)	19.6 (4.7)	3.4 (1.2)	18.1 (7.3)	7.8 (5.2)	20.9 (23.1)	41.6 (21.1)	1.3 (0.6)	0.82 (0.14)	77.3 (14.8)
	Yes	*n*	108	108	108	108	108	108	108	108	108	108
		Mean (SD)	28 (10.7)	22.7 (8.8)	3.6 (1)	17.6 (6.9)	9.6 (5.9)	26.3 (27.5)	42 (22)	1.4 (0.7)	0.68 (0.21)	68.1 (18.9)
Test (*p*-value)			T = 2.02; *p* = 0.048	T = 2.95; *p* = 0.004	T = 0.53; *p* = 0.596	T = −0.27; *p* = 0.7864	T = 1.26; *p* = 0.2103	T = 0.83; *p* = 0.4104	T = 0.08; *p* = 0.935	T = 0.66; *p* = 0.5088	T = −2.87; *p* = 0.005	T = −2.11; *p* = 0.036

**Table 6:** Exploratory multiple linear regression predicting health-related quality of life.

Predictor	B	SE B (HC3)	β	*t*	*p*	95% CI for B
Intercept	0.9966	0.134	—	7.44	<0.001	[0.731, 1.262]
BITE severity	−0.0013	0.0045	−0.03	−0.28	0.7766	[−0.010, 0.008]
Cyberbullying Index	−0.0272	0.0449	−0.08	−0.60	0.5467	[−0.116, 0.062]
IAT total	0.0005	0.0012	0.05	0.44	0.6612	[−0.002, 0.003]
Age (years)	−0.0038	0.0025	−0.15	−1.53	0.1294	[−0.009, 0.001]
BMI (kg/m^2^)	−0.0017	0.0048	−0.06	−0.35	0.7264	[−0.011, 0.008]
Comorbidity (0 = No, 1 = Yes)	−0.0850	0.0706	−0.11	−1.20	0.2313	[−0.225, 0.055]
Diagnosis: Bulimia nervosa vs. reference	−0.0259	0.0629	−0.04	−0.41	0.6816	[−0.151, 0.099]
Diagnosis: Other ED vs. reference	−0.2002	0.0897	−0.27	−2.23	0.0276	[−0.378, −0.023]
Diagnosis: Binge-eating disorder vs. reference	−0.0514	0.1616	−0.06	−0.32	0.7508	[−0.372, 0.269]

Note. Model statistics were F (9.113) = 3.45, *p* = 0.0008; R^2^ = 0.267; adjusted R^2^ = 0.221; MSE = 0.0055; RMSE = 0.074; Durbin-Watson = 1.97. SEs are heteroscedasticity-consistent (HC3). Diagnosis categories were dummy coded with anorexia nervosa as the reference group; “other ED” includes unspecified or atypical eating disorders. Comorbidity was coded as 0 = no comorbid condition (*n* = 16) and 1 = presence of any physical or mental comorbidity; due to the imbalance, this variable should be interpreted as a coarse proxy for clinical complexity.

## Data Availability

The raw data supporting the conclusions of this article will be made available by the authors on request.

## Supplementary Materials

The following supporting information can be downloaded at: https://www.mdpi.com/article/10.3390/healthcare14040476/s1, Table S1: STROBE Checklist for Cross-Sectional Studies.

## Abbreviations

The following abbreviations are used in this manuscript:

BFAS	Bergen Facebook Addiction Scale
BITE	Bulimic Investigatory Test Edinburgh
BMI	Body mass index
ED	Eating disorder
EQ-5D	EuroQol Five-Dimension Questionnaire
EQ-VAS	EuroQol Visual Analogue Scale
HRQoL	Health-related quality of life
IAT	Internet Addiction Test
SCOFF	Sick, Control, One stone, Fat, Food questionnaire
PRO	Patient-reported outcomes
VIF	Variance Inflation Factor

## Appendix A

The cyberbullying coefficient is stable in magnitude and significance across all three operationalizations, while the problematic-use indicator remains null regardless of whether it is represented by IAT, BFAS, or a composite.

**Table A1:** Sensitivity analyses for problematic online use operationalization.

Problematic-Use Variable	Cyberbullying Index B (SE HC3)	β	*p*	95% CI	Problematic-Use B (SE HC3)	β	*p*	95% CI	Adj. R^2^
IAT total	−0.1306 (0.0499)	−0.33	0.0089	[−0.2284, −0.0327]	−0.00051 (0.00131)	−0.05	0.6963	[−0.00307, 0.00205]	0.178
BFAS total	−0.1386 (0.0513)	−0.35	0.0069	[−0.2391, −0.0381]	0.00004 (0.00161)	0	0.9802	[−0.00311, 0.00319]	0.177
Composite problematic use	−0.1338 (0.0513)	−0.34	0.0091	[−0.2342, −0.0333]	−0.00727 (0.0370)	−0.03	0.8444	[−0.0799, 0.0653]	0.177

Note: Composite model: mean of z (IAT) and z (BFAS).
