# Supplementary material for: Cyberbullying and Problematic Internet Use as Correlates of Eating-Disorder Symptomatology and Health-Related Quality of Life in Women Under Specialized Care
Source: Healthcare (Basel). 2026 Feb 13;14(4):476. doi: 10.3390/healthcare14040476 (PMC12940572; doi:10.3390/healthcare14040476)
Supplement: Supplementary file 1 [file healthcare-14-00476-s001.zip › healthcare-4106135-supplementary.pdf]

Supplementary Table S1. STROBE Checklist for Cross-Sectional Studies

Item	STROBE Recommendation	Description of how addressed in the manuscript	Page/Section
**Title and Abstract**			
1a	Indicate the study's design with a commonly used term in the title or the abstract.	The title and abstract specify that this is a cross-sectional study conducted in women with eating disorders under specialized care.	Title; Abstract
1b	Provide an informative and balanced summary of what was done and what was found.	The abstract includes objectives, methods, number of participants, key results, and conclusions.	Abstract
**Introduction**			
2	Explain the scientific background and rationale for the investigation being reported.	The introduction reviews prior literature on cyberbullying, problematic internet use, eating disorder symptomatology, and HRQoL, emphasizing research gaps in clinical populations.	Section 1, Introduction
3	State specific objectives, including any prespecified hypotheses.	Three objectives and related hypotheses are explicitly presented: (1) associations between digital stressors and ED symptoms, (2) differences by comorbidity, and (3) independent predictors of HRQoL.	Section 1, Introduction
**Methods**			
4	Present key elements of study design early in the paper.	Cross-sectional analysis using baseline data from a longitudinal cohort collected between 2018 and 2019.	Section 2.1, Study Design
5	Describe the setting, locations, and relevant dates.	Data collected at a specialized eating disorder unit in Spain between January 2018 and December 2019.	Section 2.1, Setting
6a	Give the eligibility criteria and the sources and methods of selection of participants.	Inclusion: adult women with ED (anorexia, bulimia, binge-eating) receiving specialized care. Exclusion: incomplete baseline data.	Section 2.2, Participants
6b	For matched studies, give matching criteria.	Not applicable (cross-sectional design).	–
7	Clearly define all outcomes, exposures, predictors, potential confounders, and effect modifiers.	Primary outcome: BITE symptom score; Secondary: EQ-5D HRQoL; Predictors: cyberbullying index, IAT, BFAS; Covariates: age, BMI, diagnosis, comorbidity.	Section 2.3, Variables

Item	STROBE Recommendation	Description of how addressed in the manuscript	Page/Section
8	For each variable, give data sources and details of measurement.	Validated Spanish versions of SCOFF, BITE, IAT, BFAS, and EQ-5D used. Cyberbullying index adapted from prior validated items. Reliability reported.	Section 2.4, Instruments
9	Describe any efforts to address potential sources of bias.	Consecutive recruitment minimized selection bias; validated instruments minimized information bias; use of baseline data avoided attrition bias.	Section 2.5, Bias
10	Explain how the study size arrived at.	Power analysis (G*Power 3.1) indicated n=118 sufficient for medium effect (f^2=0.15, α=0.05, power=0.80). Final n=124.	Section 2.2, Sample size justification
11	Explain how quantitative variables were handled in the analyses.	Continuous variables summarized as mean (SD) or median (IQR); standardized before regression.	Section 2.6, Statistical analysis
12	Describe all statistical methods.	Non-parametric correlations, Welch's t-tests, ANOVA, and multivariable linear regression. Adjusted for age, BMI, diagnosis, and comorbidity. Diagnostics (VIF, Cook's distance) performed.	Section 2.6, Statistical analysis
**Results**			
13a	Report numbers of individuals at each stage of study.	124 participants included all baseline data complete.	Section 3.1, Participants
13b	Give reasons for non-participation.	Not applicable; all eligible participants analyzed.	–
14	Give characteristics of study participants.	Table 1 shows demographics, BMI, diagnosis, comorbidity status.	Section 3.1; Table 1
15	Report numbers of outcome events or summary measures.	Descriptive and regression results reported for BITE, IAT, BFAS, Cyberbullying, EQ-5D.	Tables 2-5
16a	Give unadjusted and adjusted estimates and precision (e.g., 95% CI).	Regression results report.	Table 5; Section 3.4
17	Report other analyses done.	Exploratory subgroup analysis by comorbidity; sensitivity checks consistent with main findings.	Section 3.5
**Discussion**			
18	Summarize key results with reference to objectives.	Sections 4.1-4.3 summarize findings consistent with stated objectives.	Discussion
19	Discuss limitations of	Limitations include cross-sectional design, single-	Section 4.4

Item	STROBE Recommendation	Description of how addressed in the manuscript	Page/Section
	the study.	center sample, self-report bias. Discussed in 4.4.	
20	Give cautious overall interpretation of results considering objectives and limitations.	Interpretation aligns findings with previous literature and acknowledges sample/contextual constraints.	Section 4.3-4.5
21	Discuss the generalisability (external validity) of the study results.	Generalizability limited to adult women in specialized care; need for multicenter replication discussed.	Section 4.4, 5
**Other information**			
22	Give the source of funding and the role of the funders.	Funded by Junta de Extremadura, with FEDER co-financing. Funders had no role in study design or interpretation.	Acknowledgments
